# Actin polymerization and longitudinal actin fibers in axon initial segment plasticity

**DOI:** 10.3389/fnmol.2024.1376997

**Published:** 2024-05-10

**Authors:** David Micinski, Pirta Hotulainen

**Affiliations:** ^1^Minerva Foundation Institute for Medical Research, Helsinki, Finland; ^2^HiLIFE-Neuroscience Center, University of Helsinki, Helsinki, Finland; ^3^Faculty of Medicine, University of Helsinki, Helsinki, Finland

**Keywords:** actin, AIS plasticity, super-resolution, axon initial segment (AIS), plasticity, formin

## Abstract

The location of the axon initial segment (AIS) at the junction between the soma and axon of neurons makes it instrumental in maintaining neural polarity and as the site for action potential generation. The AIS is also capable of large-scale relocation in an activity-dependent manner. This represents a form of homeostatic plasticity in which neurons regulate their own excitability by changing the size and/or position of the AIS. While AIS plasticity is important for proper functionality of AIS-containing neurons, the cellular and molecular mechanisms of AIS plasticity are poorly understood. Here, we analyzed changes in the AIS actin cytoskeleton during AIS plasticity using 3D structured illumination microscopy (3D-SIM). We showed that the number of longitudinal actin fibers increased transiently 3 h after plasticity induction. We further showed that actin polymerization, especially formin mediated actin polymerization, is required for AIS plasticity and formation of longitudinal actin fibers. From the formin family of proteins, Daam1 localized to the ends of longitudinal actin fibers. These results indicate that active re-organization of the actin cytoskeleton is required for proper AIS plasticity.

## Introduction

The axon initial segment (AIS) is the proximal region of the axon, about 20–40 μm in length, and it is vital for the proper functioning of the axon. This is because the AIS is where action potentials are generated, courtesy of a high degree of clustering of voltage-gated ion channels (Kole et al., 2008; Kole and Stuart, 2012; Akin et al., 2015). It is also the junction between the soma and axon. At this junction, the AIS prevents somatodendritic proteins from entering the axon and thus maintains neuronal polarity (Jones and Svitkina, 2016). Without a properly functioning AIS axonal identity can be lost, resulting in axons taking on dendritic properties and disrupting action potential firing (Hedstrom et al., 2008; Abouelezz et al., 2020). In addition to the high density of ion channels, the AIS contains scaffolding proteins and a complex cytoskeletal structure that helps to anchor the channels in place and regulate their function.

Previous work has demonstrated that the AIS is capable of large-scale reorganization and relocation in an activity-dependent manner (Grubb and Burrone, 2010; Kuba et al., 2010). This provides neurons with another opportunity to regulate and balance their output to ever-changing inputs. For example, chronic depolarization of excitatory hippocampal neurons caused a distal relocation of the AIS which decreased cell excitability (Grubb and Burrone, 2010). Long-term sensory deprivation elicited an increase in AIS length, accompanied by an increase in neuronal excitability, while sensory enrichment resulted in a rapid AIS shortening (Jamann et al., 2021). Further, dysfunctional AIS plasticity has been observed in disease models. The absence of AIS structural plasticity has been demonstrated in a frontotemporal dementia mouse model (Sohn et al., 2019).

Actin structures have been implicated in various AIS functions, including structural maintenance of the AIS, maintenance of polarity, and vesicle sorting (Al-Bassam et al., 2012; Watanabe et al., 2012; Balasanyan et al., 2017; Abouelezz et al., 2020). As some actin binding proteins, such as myosin II (Evans et al., 2017), or proteins regulating the activity of actin binding proteins, such as calcineurin (Evans et al., 2013), are necessary for the proper AIS plasticity, we hypothesized that changes in the actin cytoskeleton structures are involved in AIS plasticity.

The organization of the axonal actin cytoskeleton was first revealed when super-resolution techniques were applied to actin in neurons (Xu et al., 2013). Xu et al. showed that the axonal actin cytoskeleton is organized in evenly spaced ring-like structures (actin rings) connected to each other by spectrin. This net of actin, spectrin, and accompanying proteins is called the membrane-associated periodic skeleton (MPS). Although the MPS is best organized in axons, it is also present in dendrites and necks of dendritic spines. While actin rings are the most eye-catching actin structures in axons, there are also other types of actin structures present. In addition to actin rings, D’Este et al. (2015) observed actin bundles running along the main axis of axons. These bundles were abundant at early development (2 days *in vitro* (DIV2)) but they decreased with time. In older cultures, these bundles were seen mainly in dendrites. In a few other studies, similar bundles were found in dendrites where they were called longitudinal actin fibers (Bär et al., 2016; Konietzny et al., 2017; Lavoie-Cardinal et al., 2020). In addition to rings and longitudinal actin fibers, axons have actin patches and actin trails (Watanabe et al., 2012; Arnold and Gallo, 2014; Ganguly et al., 2015). While the organization of actin during AIS development and maintenance is largely understood, it is not known how the actin cytoskeleton changes during AIS plasticity. Thus, we wanted to analyze the changes in the actin cytoskeleton during AIS plasticity using super-resolution imaging.

We first analyzed the changes in the AIS actin cytoskeleton occurring upon chronic depolarization. Three-hour depolarization increased the number of longitudinal actin fibers transiently. We further demonstrated that actin polymerization is required for AIS plasticity, as both actin monomer sequestering and formin inhibition blocked the formation of longitudinal actin fibers and AIS plasticity. Formins are actin polymerizing factors, which polymerize straight actin filaments. We further show that one formin, Daam1, localizes to the ends of longitudinal actin fibers.

## Results

### Chronic depolarization re-organizes the actin cytoskeleton increasing the number of longitudinal actin fibers

**Figure 1:**
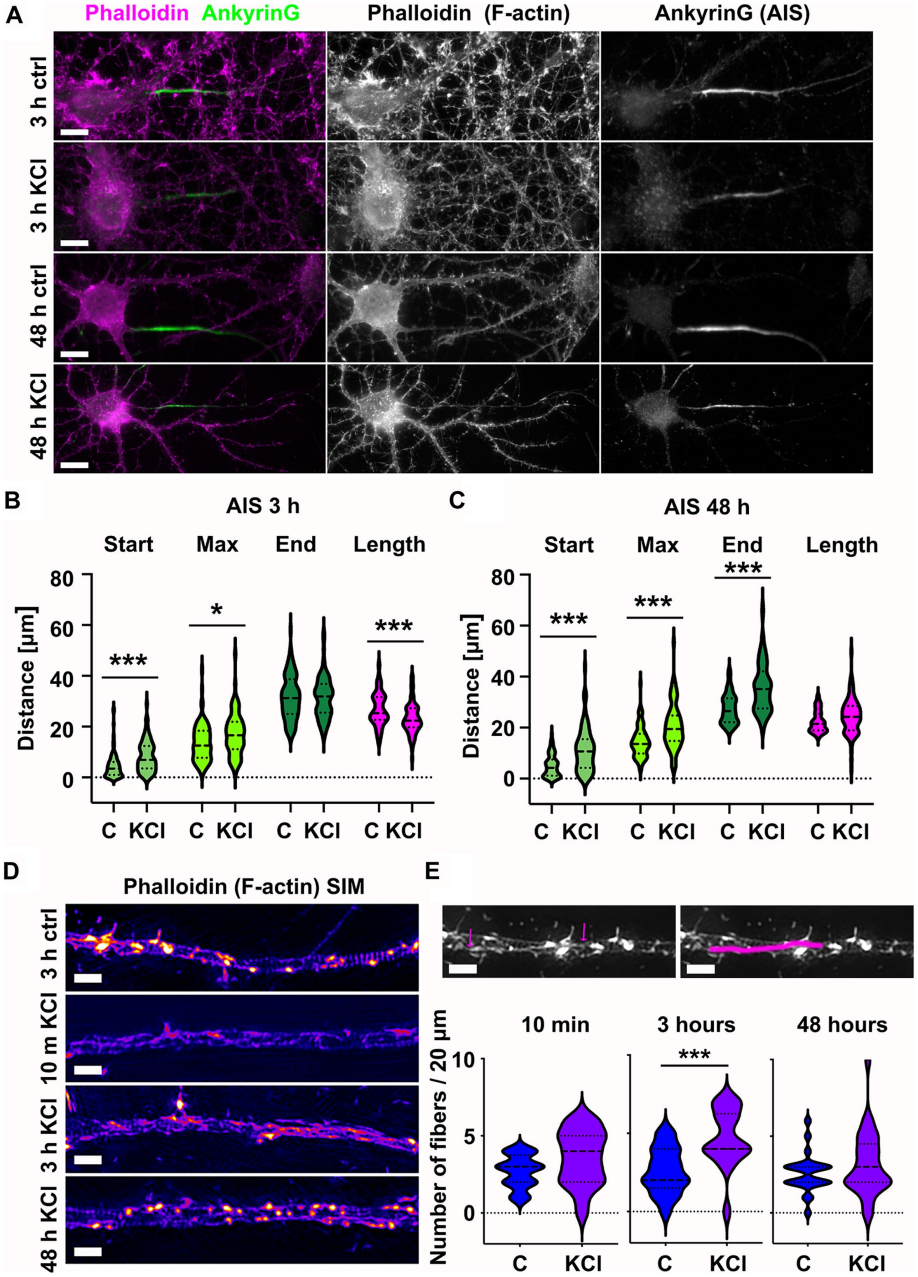
Chronic depolarization moves AIS distally and induces transient increase in longitudinal actin fibers. **(A)** DIV14 rat hippocampal neurons were treated with either 15 mM KCl to induce chronic depolarization of neurons or with 15 mM NaCl as a control. After 3-or 48-h cells were fixed and stained with phalloidin to visualize F-actin (middle column in panel, magenta on left column) and with ankyrinG antibody to visualize the AIS via ankyrinG enrichment (right column and green in left column). Scale bar: 10 μm. **(B)** The AIS was identified based on ankyrinG staining and the distance was measured from the soma or axonal branching point to the: AIS starting point (start), point of maximum ankyrinG intensity (Max), and to the end of ankyrinG staining (End). AIS length was calculated from these parameters. Data is pooled from 6 experiments and represented as mean ± SEM in μm. After 3-h KCl treatment, the average proximal end of AIS moved distally (Ctrl 4.5 ± 0.5 vs. KCl 8.3 ± 0.7) and this was seen also as a distal movement of Maximum intensity point (Ctrl 13.6 ± 0.7 vs. KCl 17.0 ± 0.9). The distal end of AIS did not move resulting in a decrease in the length of AIS (Ctrl 26.9 ± 0.7 vs. KCl 23.1 ± 0.6). For all measurements, N_ctrl_ = 96, N_KCl_ = 85. *p < 0.05 and ***p < 0.001 as determined by a Kruskal-Wallis test. **(C)** 48-h application of KCl data is pooled from 3 experiments and represented as mean ± SEM. 48-h treatment resulted in distal movement of start point (Ctrl 5.0 ± 0.5 vs. KCl 11.2 ± 0.9), maximum intensity point (Ctrl 14.5 ± 0.7 vs. KCl 20.5 ± 0.9) and the end (Ctrl 27.4 ± 0.6 vs. KCl 35.7 ± 1.0). Length of AIS was maintained. N_ctrl_ = 84, N_KCl_ = 89. ^***^p < 0.001 as determined by a Kruskal-Wallis test. **(D)** DIV14 rat hippocampal neurons were treated with 15 mM KCl or 15 mM NaCl (Ctrl) for 10 min, 3 h, or 48 h. Control is from a 3-h experiment. The AIS was identified by ankyrinG staining and approximately 20 μm length of phalloidin staining was imaged with SIM. 10-min and 3-h treatments increased the presence of longitudinal actin fibers. 48-h treated cells resembled control cells. Scale bar: 1 μm. **(E)** Longitudinal actin fibers were visually tracked and counted from 20 μm of the AIS. One example of a longitudinal actin fiber is marked to 3-h control image first with arrows and then with a line. Scale bar: 1 μm. Data is pooled from 2 (10 min) or 3 experiments (3-and 48-h) and represented as mean ± SEM. A 10-min treatment increased the number of fibers from 2.7 ± 0.2 (ctrl) to 3.6 ± 0.4 (KCl) but change was not significant. N_ctrl_ = 20, N_KCl_ = 19. 3-h treatment increased the number of fibers from 2.6 ± 0.4 (Ctrl) to 4.7 ± 0.4 (KCl). ^***^p < 0.001 as determined by a Chi-squared test. N_ctrl_ = 17, N_KCl_ = 18. 48-h treatment increased slightly the number of longitudinal actin fibers, but the change was not significant (2.6 ± 0.3 (Ctrl) to 3.2 ± 0.4 (KCl). N_ctrl_ = 24, N_KCl_ = 29. Violin plots present the median and interquartile range (25th and 75th percentile).

To study how the actin cytoskeleton changes during AIS plasticity, we induced AIS plasticity in cultured rat hippocampal pyramidal neurons by adding 15 mM KCl. Earlier studies have shown that application of 15 mM KCl induces similar changes in AIS length and location as long-term neuronal activation by optogenetics (Grubb and Burrone, 2010; Evans et al., 2015). Depolarization by 15 mM KCl produces a chronic increase in neuronal Ca^2+^-concentration and blockers of voltage-gated calcium channels (VGCCs) prevent activity-dependent AIS relocation (Grubb and Burrone, 2010). Earlier studies have further shown that KCl-treatment results in AIS shortening in 3 h (Evans et al., 2015), and distal translocation of the entire AIS in 48-h (Grubb and Burrone, 2010; Evans et al., 2013, 2017). Following these approaches, we imaged the location and length of AIS structures after 3 h-and 48 h-KCl-treatments and compared images to controls treated with NaCl (Figure 1A). Phalloidin staining, visualizing the filamentous actin (F-actin), was used to visualize cell morphology. Organization of the actin cytoskeleton, especially in dendrites, is a good parameter to estimate the health of neurons after relatively harsh KCl treatments (Figure 1A). The AIS was labeled with ankyrinG, which is enriched in the AIS thus providing a clear marker for the AIS. AnkyrinG is a scaffolding protein which plays a crucial role at the structural and functional levels. AnkyrinG loss leads to AIS disassembly and consequently the loss of neuronal polarity (Hedstrom et al., 2008; Sobotzik et al., 2009).

We tracked the AIS based on ankyrinG staining using custom MATLAB scripts from Van Beuningen et al. (2015).^1^ This script identifies the brightest area (maximum) of the ankyrinG staining and measures the start and end points of the AIS as the points on either end of the maximum where fluorescence intensity drops to 33% of max. In control conditions, the AIS starts close to the soma or a branch where the axon starts. After 3-h-KCl-treatment, the AIS start point was on average 4 μm further along the axon and AISes were on average 4 μm shorter than AISes in control conditions (Figure 1B). After 48 h, both the “start” and the “end” moved distally 6 and 9 μm, while the length was maintained (Figure 1C).

Primary hippocampal neuron cultures contain different cell types. In contrast to excitatory neurons, inhibitory neurons do not express KCl-induced AIS translocation (Grubb and Burrone, 2010). Thus, we avoided analyzing inhibitory neurons. Inhibitory neurons were distinguished from excitatory neurons based on Gad65/67-expression and F-actin phenotype. To determine whether KCl-treatment reduces the concentration of ankyrinG in the AIS, we measured the relative intensity of the ankyrinG staining in the AIS versus dendrites within each neuron. This analysis revealed that there was no difference in relative ankyrinG staining intensity between different treatments (data not shown).

While we know how the actin cytoskeleton responds to neuronal activation in dendrites or in dendritic spines (Hlushchenko et al., 2016; Lavoie-Cardinal et al., 2020), we have relatively poor knowledge on activity-induced changes in the axonal actin cytoskeleton. Compared to the dendritic actin cytoskeleton, the AIS actin cytoskeleton is dim and no clear actin structures can be identified by using traditional techniques of light microscopy (see Figure 1A). Therefore, we imaged the fine structures of the actin cytoskeleton using the structured illumination microscopy (SIM) super-resolution technique (Figure 1D). SIM imaging revealed that AIS areas had more longitudinal actin fibers after 3-h KCl treatment. To measure the increase, we manually tracked longitudinal actin fibers from images (an example shown in Figure 1E), analyzing 20 μm length of each AIS. By going up and down in the SIM Z-stack, we ensured that all tracked fibers were inside the axon. This image analysis showed that the number of longitudinal actin fibers almost doubled from 2.6 to 4.7 per 20 μm of analyzed axon length after 3 h of KCl-treatment (Figure 1E). After 48 h, the number of longitudinal actin fibers was back to control values and visually, the AIS actin cytoskeleton resembled the control condition. Based on these results, it seems that the actin cytoskeleton re-organizes upon KCl-treatment in hours but then reverts back to normal in 48-h. We were curious to know how fast changes in the actin cytoskeleton occur and therefore we also examined actin fibers at an additional 10-min timepoint. Although there was no significant change, the increase in longitudinal actin fibers was visible already at 10 min (Figures 1D,E). From these experiments we can conclude that increase in longitudinal actin fibers begins rapidly after adding KCl, reaching maximal change in hours. Change is, however, transient and in 48-h, the AIS actin cytoskeleton resembles control conditions again.

**Figure 2:**
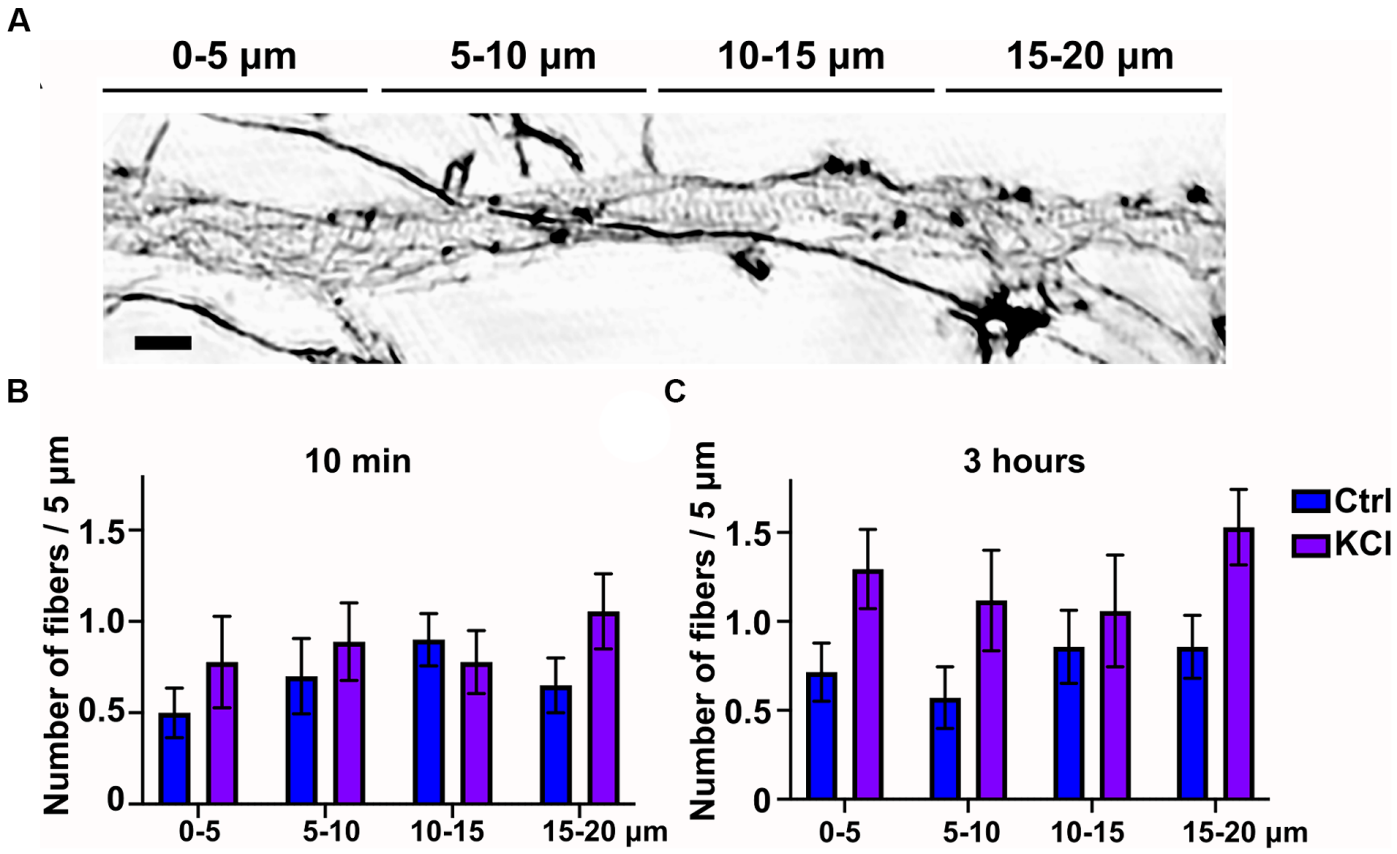
Number of longitudinal actin fibers increases througout the AIS. **(A)** DIV14 rat hippocampal neurons were treated with 15 mM KCl or 15 mM NaCl (Ctrl) for 10 min or 3 h. Image shown is a neuron treated with KCl for 10 min. The AIS was identified by ankyrinG staining and approximately 20 μm was imaged with SIM. For spatial analysis this 20 μm was divided to four areas of 5 μm. Scale bar: 1 μm. **(B,C)** Data collected for Figure 1E was re-analyzed by specifying the area where each longitudinal actin fiber located. Data is pooled from 2 (10 min) or 3 experiments (3-h) and represented as mean ± SEM. KCl treatment increased the number of longitudinal actin fibers but no change reached statistical significance determined by a Chi-squared test.

Since 3-h KCl-treatment shortens the AIS from the proximal end, we hypothesized that changes in the actin cytoskeleton take place primarily in the proximal end of the AIS. Thus, we analyzed whether we could observe spatial changes along the AIS in longitudinal actin fibers in KCl treated cells compared to NaCl treated control cells. We split our 20 μm images of the AIS to 4 areas of 5 μm, organized from proximal to distal ends of the AIS. This 20 μm area includes nearly the entire AIS, as the average length in our experiments was just slightly over 20 μm. The example image shows AIS phalloidin staining in a neuron treated with KCl for 10 min (Figure 2A). This image is brightened and gamma-corrected to make different structures clearly visible. SIM works by using patterned illumination, usually stripes, to excite the sample. The stripe position and orientation are changed several times, and the emitted fluorescence signal is recorded for each of those positions. Brightening of figures sometimes makes these stripes visible as imaging artefacts, as seen in the background of this image. Spatial analysis of 10-min and 3-h timepoints showed that KCl-treatment increased longitudinal actin fibers relatively equally, with no area appearing to be favored over another (Figures 2B,C). Live-cell imaging may reveal greater polymerization of fibers in particular spatial domains earlier or later than the observed timepoints, however the current analysis shows our original hypothesis to be incorrect with regard to longitudinal actin fibers.

Taken together, KCl treatment induces translocation of the AIS. Translocation begins with shortening of the AIS from the proximal end in hours. This step is accompanied by changes in the actin cytoskeleton, characterized by an increase in longitudinal actin fibers. After two days of KCl treatment, both the proximal and distal ends of the AIS moved distally, while AIS length was maintained. At this time point, the AIS actin cytoskeleton was indistinguishable from the controls.

### Longitudinal actin fibers are present also in distal axons

**Figure 3:**
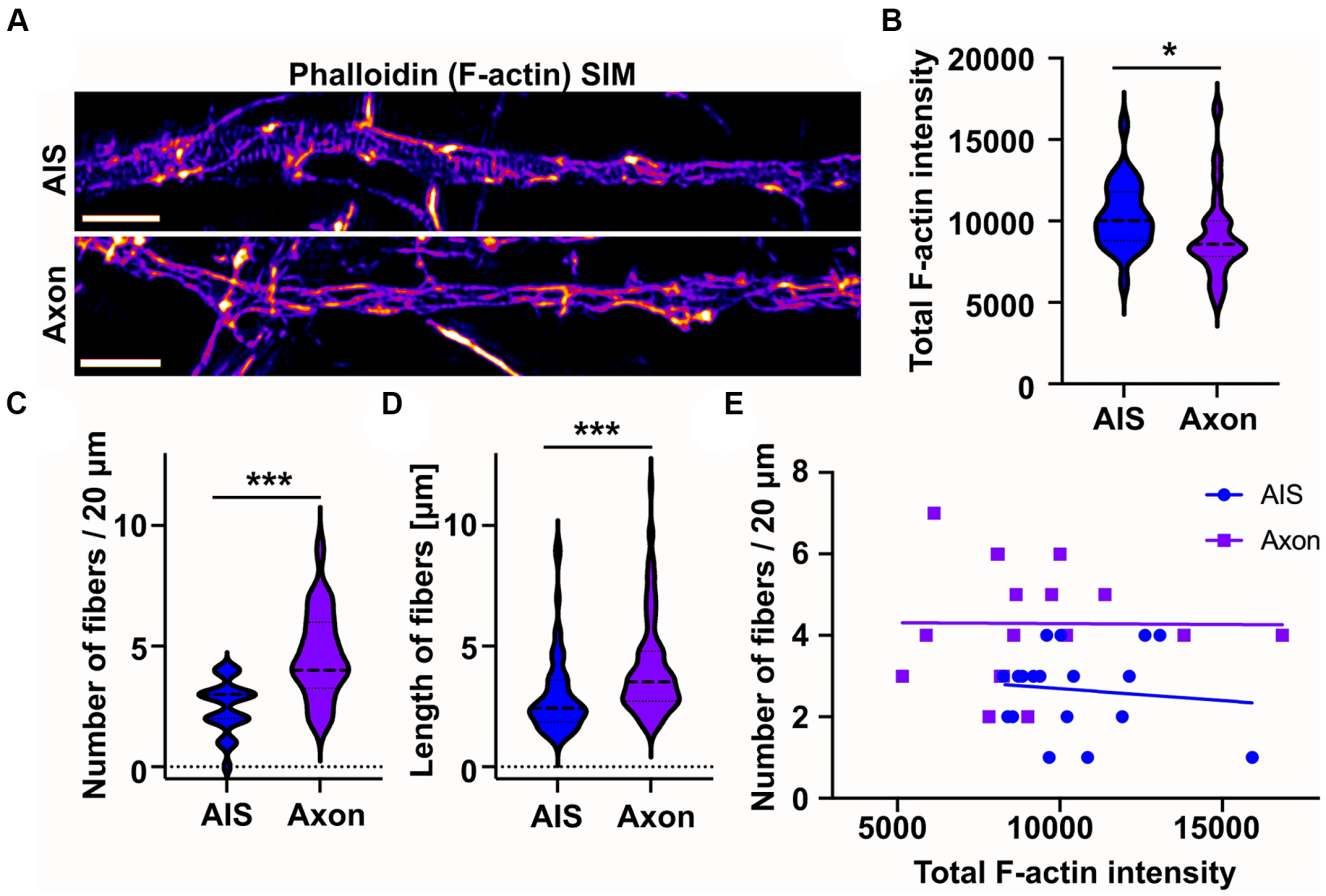
Longitudinal actin fibers are shorter in the AIS than in the distal axon. **(A)** DIV14 rat hippocampal neurons were fixed and stained with phalloidin to visualize F-actin and with ankyrinG antibody to visualize AIS area. Then 20 μm length from the AIS and distal axon were imaged with SIM. Scale bar: 1 μm. **(B)** Total F-actin intensity was measured from the image areas used for further analyses and mean values of each area were plotted. Data is pooled from 3 experiments and represented as mean ± SEM. Average mean values for AIS images was 10,333 ± 408 and for distal axon 9,105 ± 551 (arbitrary units). N_AIS_ = 25, N_AXON_ = 23. *p < 0.05 as determined by a Mann–Whitney U test. **(C)** Longitudinal fibers were visually tracked and the number and length of individual fibers were measured using Fiji from 20 μm of both the AIS and distal axon. Data is pooled from 3 experiments and represented as mean ± SEM. Number of longitudinal actin fibers in the analyzed 20 μm area was 2.5 ± 0.2 in the AIS area and 4.6 ± 0.4 in the distal axon. N_AIS_ = 24, N_AXON_ = 24. ^***^p < 0.001 as determined by a Chi-squared test. **(D)** Length of longitudinal actin fibers was 3.0 ± 0.2 μm in the AIS and 4.1 ± 0.2 μm in the distal axon. Data is pooled from 3 experiments. N_AIS_ = 60, N_AXON_ = 111. ^***^p < 0.001 as determined by a Mann–Whitney U test. **(E)** The number of longitudinal actin fibers of each AIS or distal axon was plotted based on measured total F-actin intensity to test whether there is a correlation between them. Values from only two experiments were plotted to keep the clarity. No correlation between tested parameters was detected. Violin plots present the median and interquartile range (25th and 75th percentile).

From earlier literature we knew that longitudinal actin fibers are present in dendrites and now we have observed them in the AIS. However, there is no direct information on whether longitudinal actin fibers are also present in distal axons. Therefore, in addition to the AIS, we imaged areas from distal axons beyond ankyrinG staining. This approach revealed that there are longitudinal actin fibers also in distal axons (Figure 3A). To test whether intensity of phalloidin staining is comparable in the images taken from the AIS and distal axon, we measured the total F-actin intensity of SIM images. The average F-actin intensities were slightly higher in AIS images compared to distal axon images (Figure 3B). We then measured the number and length of longitudinal actin fibers in the AIS and distal axon and found that there are more longitudinal actin fibers in the distal axons than in the AIS in control conditions (Figure 3C). Longitudinal actin fibers were also 1 μm longer in distal axons compared to AIS area (Figure 3D). To test whether the number of longitudinal actin fibers depends on the general brightness of imaged filamentous actin, we analyzed whether there is any correlation between the total F-actin intensity (Figure 3B) and number of longitudinal actin fibers (Figure 3C), but there was no such correlation (Figure 3E).

These results show that longitudinal actin fibers are not specific to the AIS but are present also in distal axons.

### Actin polymerization is required for AIS plasticity

**Figure 4:**
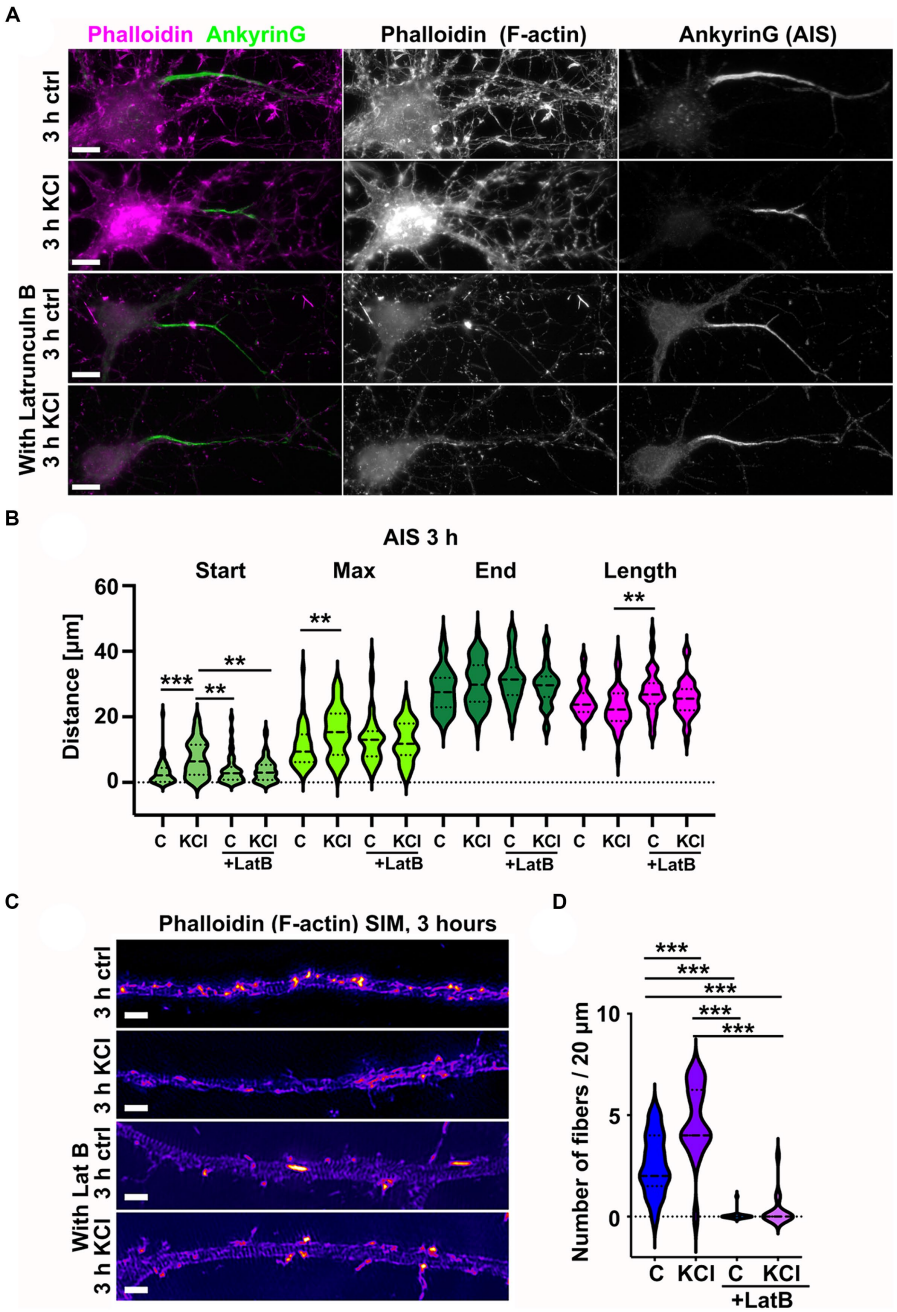
Latrunculin B treatment blocks AIS plasticity and formation of longitudinal actin fibers. **(A)** DIV14 rat hippocampal neurons were treated with either 15 mM KCl to induce chronic depolarization of neurons or with 15 mM NaCl as a control in the presence or absence of 5 μM latrunculin B. After 3 h, cells were fixed and stained with phalloidin to visualize F-actin (middle column in panel, magenta on left column) and with ankyrinG antibody to visualize the AIS by ankyrinG enrichment (right column and green in left column). Scale bar: 10 μm. **(B)** The AIS was identified based on ankyrinG staining and the distance was measured from the soma or axonal branching point to the: AIS starting point (Start), point of maximum ankyrinG intensity (Max), and to the end of ankyrinG staining (End). AIS length was calculated from these parameters. Data is pooled from three experiments and represented as mean ± SEM in μm. After 3-h KCl treatment, the average proximal end of AIS moved distally without latrunculin B but in the presence of latrunculin B, this movement did not occur (Ctrl 3.1 ± 0.6; KCl 7.3 ± 0.8; LatB 3.8 ± 0.6; LatB+KCl 3.8 ± 0.5). Similarly, the maximum intensity point moved distally in KCl treatment but not in the presence of Latrunculin B (Max: Ctrl 10.9 ± 1.0; KCl 15.7 ± 1.0; LatB 13.2 ± 1.1; LatB+KCl 12.7 ± 0.9) (End: Ctrl 28.1 ± 1.0; KCl 30.3 ± 1.0; LatB 31.3 ± 1.0; LatB+KCl 29.4 ± 0.9) (Length: Ctrl 25.0 ± 0.8; KCl 23.0 ± 0.9; LatB 27.5 ± 1.0; LatB+KCl 25.6 ± 0.8). For all measurements, N_ctrl_ = 43, N_KCl_ = 50, N_LatB_ = 45, N_LatB + KCl_ = 48. **p < 0.01, ***p < 0.001 as determined by a Kruskal-Wallis test. **(C)** DIV14 rat hippocampal neurons were treated with 15 mM KCl or 15 mM NaCl (Ctrl) for 3 h with or without latrunculin B. The AIS was identified by ankyrinG staining and approximately 20 μm length of phalloidin staining was imaged with SIM. The 3-h KCl treatment increased the presence of longitudinal actin fibers without latrunculin B, but latrunculin B inhibited formation of longitudinal actin fibers. Scale bar: 1 μm. **(D)** Longitudinal actin fibers were visually tracked and counted from 20 μm length of the AIS. Data is pooled from 3 experiments and represented as mean ± SEM. 3-h KCl treatment increased the number of fibers from 2.6 ± 0.4 (ctrl) to 4.7 ± 0.4 (KCl). In the presence of latrunculin B, very few cells had any fibers, and this did not change upon KCl addition (0.1 ± 0.1 (LatB) to 0.3 ± 0.2 (LatB+KCl)). N_ctrl_ = 17, N_KCl_ = 18, N_LatBl_ = 18, N_LatB + KCl_ = 16. ^***^p < 0.001 as determined by a Chi-squared test. Violin plots present the median and interquartile range (25th and 75th percentile).

As KCl-treatment induced the appearance of new actin filament structures in the AIS, we wanted to test whether actin filament polymerization is necessary for AIS plasticity. Latrunculin binds monomeric actin and alters the actin-monomer subunit interface to prevent polymerization (Morton et al., 2000). This sequesters free actin monomers, thus blocking polymerization of actin filaments. We repeated the 3-h KCl treatment with latrunculin B to see whether blocking actin polymerization with latrunculin B would also block shortening of the AIS (Figure 4A). Analysis of AIS location and length revealed that the AIS length was not reduced in the presence of latrunculin B (Figure 4B). As actin polymerization is crucial for the maintenance of neuron morphology in general, we did not do a 48-h KCl treatment with latrunculin B. Phalloidin staining shows how actin is depolymerized from the dendrites and soma area in 3 h. However, axons are stable and visually appear mostly intact (Figures 4A,B).

We then analyzed the fine structures of the AIS actin cytoskeleton using SIM. In the presence of latrunculin B, longitudinal actin fibers were lost both from NaCl (control)- and KCl-treated neurons. The fact that latrunculin B can dissolve longitudinal actin fibers suggests that they are continuously depolymerized and polymerized (Figures 4C,D).

These results show that latrunculin B treatment led to depolymerization of longitudinal actin fibers and blocked the AIS plasticity induced by 3-h KCl treatment.

### KCl-treatment has a modest effect on actin ring periodicity

**Figure 5:**
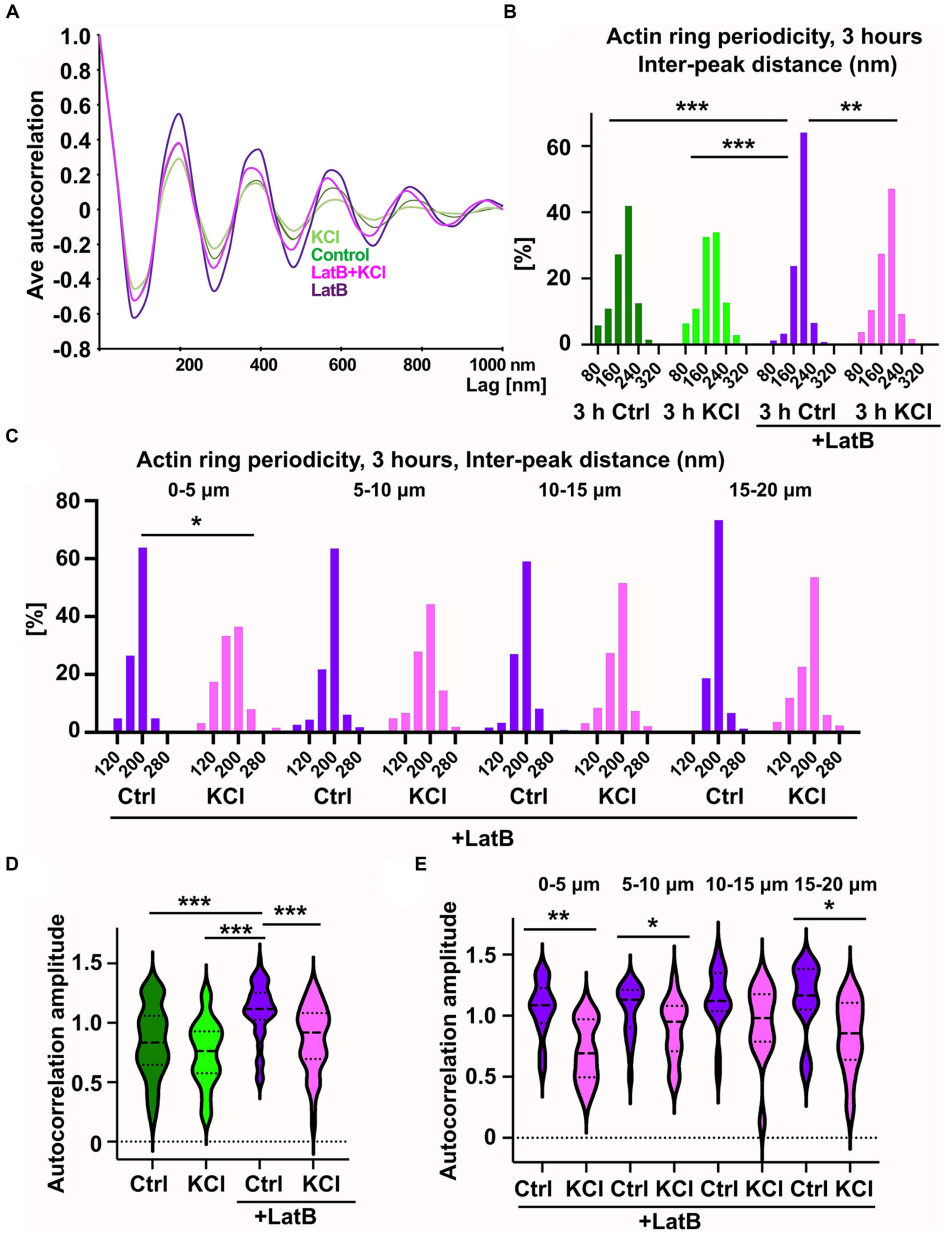
KCl-treatment has a modest effect on actin ring periodicity. **(A)** Autocorrelation analysis of F-actin distributions of NaCl (Ctrl), KCl, latrunculin B, and latrunculin B and KCl treated neurons (3-h treatment). Shown are the averaged autocorrelation from multiple segments of AIS for each condition. **(B)** The distance between adjacent peaks in all fluorescence intensity profiles was measured and distributions of inter-peak distances were calculated. Distribution of LatB treated cells differed from others significantly as determined by a Kolmogorov–Smirnov test, ^**^p < 0.01, ^***^p < 0.001. Data is pooled from 3 (LatB) and 4 (ctrl) experiments and N_ctrl_ = 37, N_KCl_ = 30, N_LatBl_ = 18, N_LatB + KCl_ = 16. **(C)** Spatial analysis of NaCl-and KCl-treated cells in the presence of latrunculin B shows that frequencies change significantly at the most proximal area of the AIS as determined by a Kolmogorov–Smirnov test, *p < 0.05. Data is pooled from 3 experiments having altogether 18 (LatB) and 16 (LatB+KCl) cells, and four measured areas in each cell. The most proximal area has 15 (LatB) and 12 (LatB+KCl) analyzed areas. **(D)** The average amplitude of autocorrelation analysis for different treatments (ctrl, KCl, Latrunculin B with and without KCl). The amplitude was measured as the difference between the first peak and the average of the first two valleys of the autocorrelation curve. Amplitudes of latrunculin B treated cells differ from amplitudes in other treatments as determined by a Kruskal-Wallis test, ^***^p < 0.001. Data is pooled from 3 (LatB) and 2 (ctrl) experiments having altogether 15 (Ctrl), 15 (KCl), 17 (LatB) and 14 (LatB+KCl) cells and 47 (Ctrl), 51 (KCl), 67 (LatB) and 51 (LatB+KCl) areas. **(E)** Spatial analysis of NaCl-and KCl-treated cells in the presence of latrunculin B shows that amplitude change significantly at three areas as determined by Mann–Whitney U test, ^*^p < 0.05, ^**^ < 0.01. Data is pooled from 3 experiments having altogether 17 (LatB) and 14 (LatB+KCl) cells. The most proximal area has 14 (LatB) and 8 (LatB+KCl) analyzed autocorrelation curves, second area has 21 and 16, third area 20 and 15, and fourth area 12 and 12, respectively. Violin plots present the median and interquartile range (25th and 75th percentile).

An earlier study showed that 10-min neuronal activation re-organized actin rings into longitudinal fibers in dendrites but not in axons (Lavoie-Cardinal et al., 2020). Since we detected an increase in longitudinal actin fibers upon three-hour KCl-treatment (Figure 1), we examined whether we can also detect changes in actin rings. Actin rings were found in all treatment conditions (Figure 4C). As seen before (Leterrier et al., 2015; Abouelezz et al., 2019), actin rings were especially clear in the AIS area after latrunculin B treatment. To analyze changes in actin rings, we used the periodicity analysis that we had used in our earlier studies (Abouelezz et al., 2019, 2020). The SIM camera used has a 40 nm pixel size and therefore all distances between adjacent peaks are multiples of 40 nms. The average autocorrelation curves for all groups showed significant autocorrelation at a lag of 200 nm (Figure 5A). Latrunculin treated neurons showed clear improvement in periodicity accuracy, with the distribution shifted towards enrichment of 200 nm peaks (Figure 5B). We think that this shift in distribution is mainly due to the loss of longitudinal actin fibers, as these fibers may interfere with the analysis of periodicity by partially occluding actin rings. In contrast to dynamic fibers, the actin filaments in rings have such a low turnover of actin monomers, low depolymerization and polymerization, that sequestering monomers with latrunculin B has no effect on them.

Without latrunculin B, 3-h KCl treatment slightly reduced the enrichment of peaks at 200 nm but there was no change in the total distribution of peaks compared to control neurons (Figure 5B). However, when “periodicity clarity” was improved with latrunculin B treatment, there was a significant difference between the peak distributions, with a substantial decrease in peaks at 200 nm in KCl-treated neurons (Figure 5B). We further analyzed the data testing whether the overall proportion of peaks specifically at 200 nm is different for different treatments. The proportion of peaks at 200 nm was lower in cells treated with both latrunculin B and KCl (47%) compared to cells treated only with latrunculin B (64%) (*p* < 0.001, 2-sample test for equality of proportions with continuity correction). Similarly, proportion was significantly lower in KCl-treated cells (38%) compared to control cells (48%) (*p* < 0.01, 2-sample test for equality of proportions with continuity correction).

We tested our hypothesis that changes in the actin cytoskeleton take place primarily in the proximal end of AIS also with periodicity analysis. Spatial analysis of the distributions of interpeak distances (four 5 μm areas instead of full 20 μm length) showed that, in the presence of latrunculin B, 3-h KCl-treatment reduced the peaks at 200 nm especially in the proximal end of the AIS, suggesting that actin dynamics can vary between different areas in the AIS during AIS plasticity. Also without latrunculin B, when control and KCl-treated cells were compared, the most proximal end showed clearest, but not significant, difference. We further analyzed the data testing whether the proportion of peaks at 200 nm is different for different treatments and areas. The proportion of peaks at 200 nm was lower in cells treated with latrunculin B and KCl (37%) compared to cell treated only with latrunculin B (64%) at most proximal end of AIS (p < 0.01, 2-sample test for equality of proportions with continuity correction). Areas between 5–10 μm and 15–20 μm also showed a significant change in the proportion of 200 nm peaks using this test (p < 0.01 and *p* < 0.05, respectively). The proportion of 200 nm peaks was also significantly lower in KCl-treated cells (30%) compared to control cells (47%) in the most proximal end of AIS (*p* < 0.01, 2-sample test for equality of proportions with continuity correction) and in the next most proximal area at 5–10 μm (Ctrl: 44% vs. KCl: 32%, *p* < 0.05).

We continued by calculating the amplitude of autocorrelation curves for different treatments. The amplitude was measured as the difference between the first peak and the average of the two first valleys of the autocorrelation curve as in Zhong et al. (2014). Average autocorrelation curves and the amplitude analysis of each analyzed line showed that latrunculin B treated cells had largest amplitude and amplitude decreased with 3-h KCl-treatment (Figures 5A,D). We further analyzed the spatial changes for every 5 μm along the AIS length. Spatial analysis revealed that amplitude changed mostly at the proximal end of the AIS (Figure 5E).

These results show that KCl has an effect on actin ring periodicity in the AIS. It is important to note that latrunculin B treatment blocked AIS shortening even in the presence of KCl. Thus, while this change in periodicity was not sufficient for plasticity induced AIS shortening, it may be a necessary process for the changes in AIS location. In general, the effect of neuronal activation on actin ring periodicity was relatively modest in axons when it is compared to the effect on dendrites (Lavoie-Cardinal et al., 2020).

### Formin activity is required for AIS plasticity

**Figure 6:**
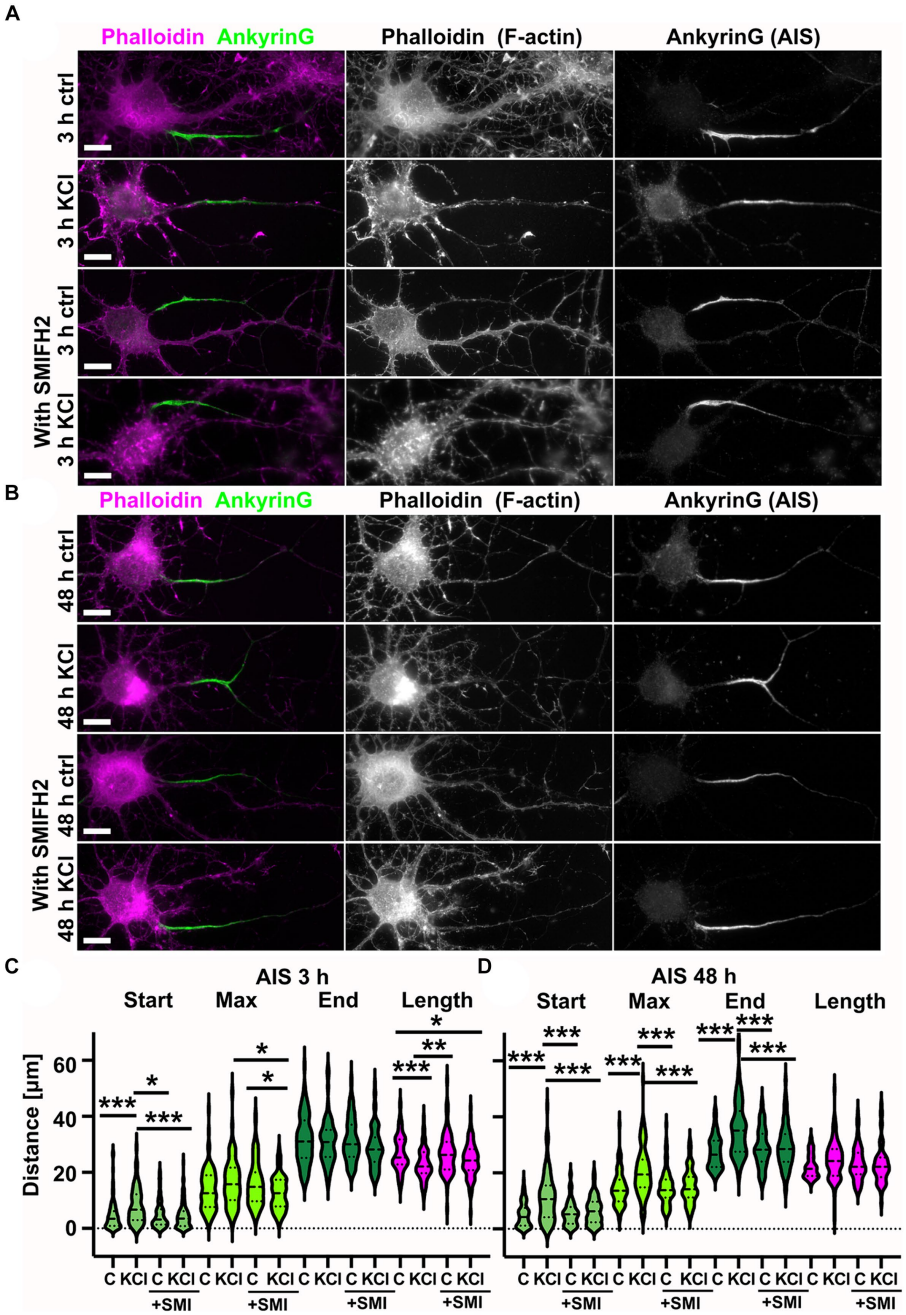
SMIFH2 formin inhibitor abolished chronic depolarization-induced AIS relocation. **(A,B)** DIV14 rat hippocampal neurons were treated with either 15 mM KCl to induce chronic depolarization of neurons or with 15 mM NaCl as a control in the presence or absence of 10 μM SMIFH2. After 3 h **(A)** or 48-h **(B)** cells were fixed and stained with phalloidin to visualize F-actin (middle column in panel, magenta on left column) and with ankyrinG antibody to visualize AIS area by ankyrinG enrichment (right column and green in left column). Scale bar: 10 μm. **(C)** The AIS was identified based on ankyrinG staining and the distance was measured from the soma or axonal branching point to the: AIS starting point (Start), point of maximum ankyrinG intensity (Max), and to the end of ankyrinG staining (End). AIS length was calculated from these parameters. Data for the three-hour timepoint is pooled from five experiments and represented as mean ± SEM in μm. ^*^p < 0.05, ^**^p < 0.01, ^***^p < 0.001 as determined by a Kruskal-Wallis test. After 3-h KCl treatment, the average proximal end of the AIS moved distally without SMIFH2 but in the presence of SMIFH2, this movement did not occur (Ctrl 4.6 ± 0.6; KCl 8.0 ± 0.8; SMIFH2 4.9 ± 0.5; SMIFH2 + KCl 4.4 ± 0.4)(Max: Ctrl 13.8 ± 0.8; KCl 16.4 ± 1.0; SMIFH2 16.0 ± 0.8; SMIFH2 + KCl 12.7 ± 0.6)(End: Ctrl 31.6 ± 0.9; KCl 31.0 ± 1.0; SMIFH2 31.6 ± 0.8; SMIFH2 + KCl 28.7 ± 0.7). The length of AIS shortened with KCl (Ctrl 27.0 ± 0.7; KCl 23.0 ± 0.7; SMIFH2 26.7 ± 0.7; SMIFH2 + KCl 24.3 ± 0.6). For all measurements, N_ctrl_ = 87, N_KCl_ = 69, N_SMIFH2_ = 90, N_SMIFH2 + KCl_ = 99. **(D)** Data for the 48-h timepoint is pooled from 3 experiments and represented as mean ± SEM. ^***^p < 0.001 as determined by a Kruskal-Wallis test. After 48-h KCl treatment, the proximal end, maximum intensity point, and end point of the AIS moved distally without SMIFH2 but in the presence of SMIFH2, this movement did not occur (Ctrl 5.0 ± 0.5; KCl 11.2 ± 0.9; SMIFH2 5.6 ± 0.5; SMIFH2 + KCl 6.5 ± 0.6)(Max: Ctrl 14.5 ± 0.7; KCl 20.5 ± 0.9; SMIFH2 14.7 ± 0.7; SMIFH2 + KCl 15.2 ± 0.7)(End: Ctrl 27.4 ± 0.6; KCl 35.7 ± 1.0; SMIFH2 28.9 ± 0.7; SMIFH2 + KCl 29.4 ± 1.0). The length of AIS did not change in any treatment (Ctrl 22.4 ± 0.4; KCl 24.5 ± 0.7; SMIFH2 23.3 ± 0.7; SMIFH2 + KCl 22.9 ± 0.8). For all measurements, N_ctrl_ = 84, N_KCl_ = 89, N_SMIFH2_ = 85, N_SMIFH2 + KCl_ = 67. Violin plots present the median and interquartile range (25th and 75th percentile).

As longitudinal actin fibers resemble other actin structures polymerized by proteins belonging to the formin family, we next tested whether the general formin inhibitor SMIFH2 affects AIS plasticity. We treated hippocampal neurons with 15 mM KCl to induce AIS plasticity with or without 10 μM SMIFH2 for 3 h and 48 h (Figures 6A,B). SMIFH2 treatment blocked AIS plasticity at both time points (Figures 6C,D).

**Figure 7:**
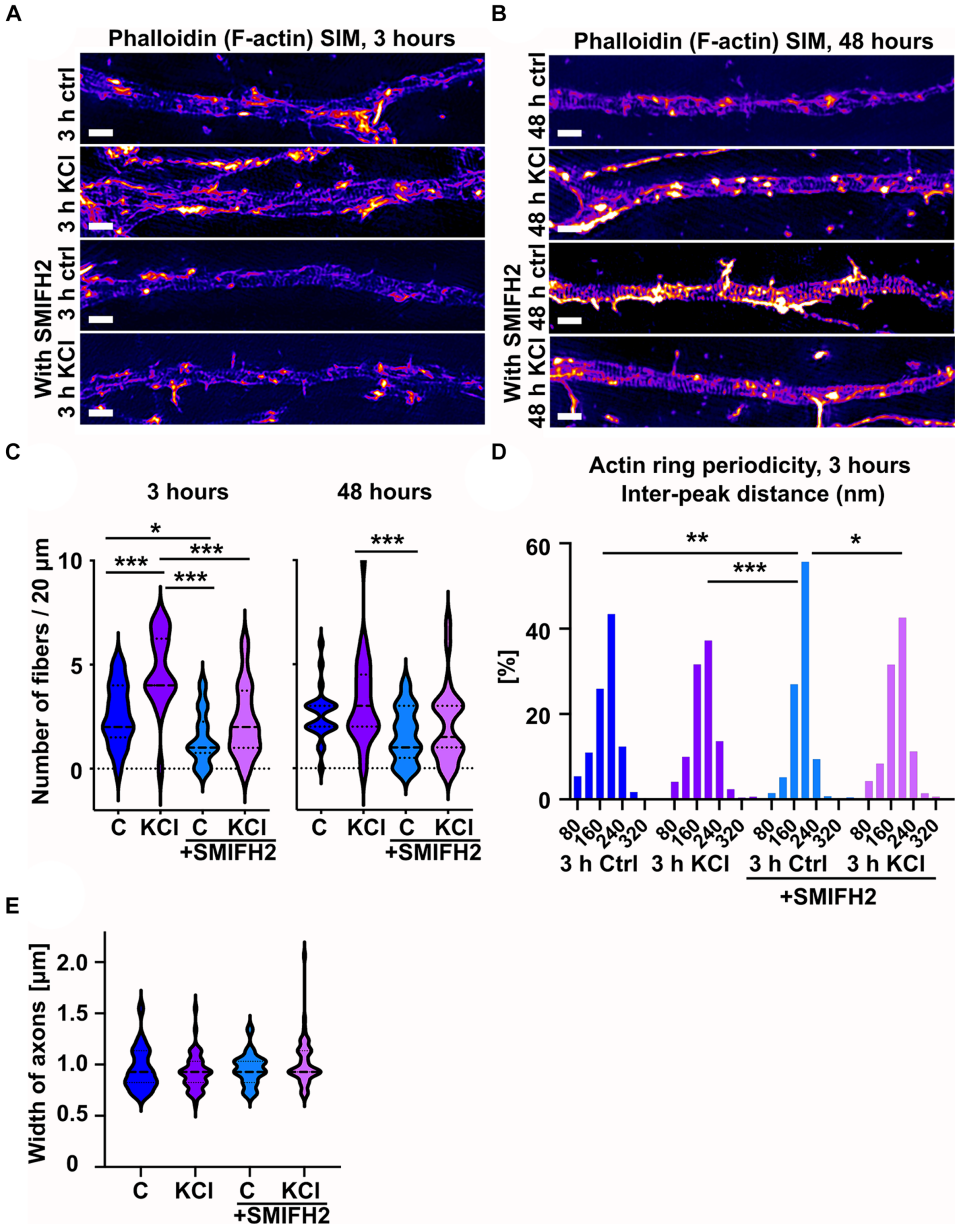
SMIFH2 treatment reduces the number of longitudinal actin fibers. **(A,B)** DIV14 rat hippocampal neurons were treated with 15 mM KCl or 15 mM NaCl (Ctrl) for 3 h **(A)** or 48 h **(B)** with or without SMIFH2. The AIS was identified by ankyrinG staining and approximately 20 μm length of phalloidin staining was imaged with SIM. 3-h KCl treatment increased the presence of longitudinal actin fibers without SMIFH2 but SMIFH2 inhibited formation of longitudinal actin fibers. 48-h long KCl-treatment did not increase the presence of longitudinal actin fibers but SMIFH2 treatment seemed to reduce them. Scale bar: 1 μm. **(C)** Longitudinal actin fibers were visually tracked and counted from 20 μm length of the AIS. Data is pooled from 3 experiments and represented as mean ± SEM. 3-h KCl treatment increased the number of fibers from 2.6 ± 0.4 (ctrl) to 4.7 ± 0.4 (KCl). In the presence of SMIFH2, the number of fibers was reduced in both ctrl and with KCl (1.5 ± 0.3 (SMIFH2), 2.3 ± 0.3 (SMIFH2 + KCl)). N_ctrl_ = 17, N_KCl_ = 18, N_SMIFH2_ = 22, N_SMIFH2 + KCl_ = 28. 48-h KCl treatment did not significantly increase the number of longitudinal actin fibers. SMIFH2 treatment decreased the number of fibers when compared to KCl-treatment (2.6 ± 0.3 (ctrl), 3.2 ± 0.4 (KCl), 1.6 ± 0.3 (SMIFH2), 2.1 ± 0.4 (SMIFH2 + KCl)). N_ctrl_ = 24, N_KCl_ = 29, N_SMIFH2_ = 25, N_SMIFH2 + KCl_ = 22. ^*^p < 0.05, ^***^p < 0.001 as determined by a Chi-squared test. **(D)** The distance between adjacent peaks in all fluorescence intensity profiles was measured and distributions of inter-peak distances were calculated. Data is pooled from 4 experiments for control and KCl-treated neurons and from 3 experiments in both SMIFH2-treated conditions. The distribution of SMIFH2 treated cells differed from others significantly as determined by a Kolmogorov–Smirnov test. N_ctrl_ = 37, N_KCl_ = 30, N_SMIFH2_ = 20, N_SMIFH2 + KCl_ = 24. ^*^p < 0.05, ^**^p < 0.01, ^***^p < 0.001. **(E)** The width of analyzed axons was measured from two experiments of 48-h-treatments. The width of axons was similar in controls and SMIFH2 treated cells (0.9–1.0 μm). N_ctrl_ = 45, N_KCl_ = 51, N_SMIFH2_ = 37, N_SMIFH2 + KCl_ = 48. Violin plots present the median and interquartile range (25th and 75th percentile).

Analysis of actin organization using SIM revealed that SMIFH2 treatment reduced the number of longitudinal actin fibers (Figures 7A,B). Three-hour-KCl treatment slightly increased the number of longitudinal actin fibers even in the presence of SMIFH2 but it could not increase the number of longitudinal actin fibers above control conditions (Figure 7C). We also analyzed the periodicity of actin rings, which revealed that SMIFH2 treatment had a similar effect as latrunculin B on periodicity when compared to control (Figure 7D). SMIFH2 treatment shifted the distribution towards enrichment of 200 nm peaks. Similar to what was observed with latrunculin B treatment, improved “periodicity clarity” with SMIFH2 resulted in significantly fewer peaks at 200 nm in cells treated with SMIFH2 and KCl compared to cells treated with SMIFH2 alone (Figure 7D).

SMIFH2 also inhibits myosin II in higher concentrations and when long treatments are used. However, the concentration used here (10 μM) reduces myosin II activity by only 10% according to *in vitro* analysis (Nishimura et al., 2021). This concentration reduced the actin assembly rate of various formins by approximately 80% (Rizvi et al., 2009). In earlier publications, myosin II inhibition was shown to increase axonal diameter (Costa et al., 2020; Wang et al., 2020). Thus, to check for possible myosin II inhibition, we measured the width of analyzed axons in the area of the AIS. Axon width was measured approximately from the same distance from the soma to avoid any differences that might arise due to measuring the axon at varying distances from the soma. The mean width of axons was similar in controls and SMIFH2 treated cells suggesting that myosin II was not significantly affected (Figure 7E).

### Formin family protein Daam1 localizes to the ends of longitudinal actin fibers

**Figure 8:**
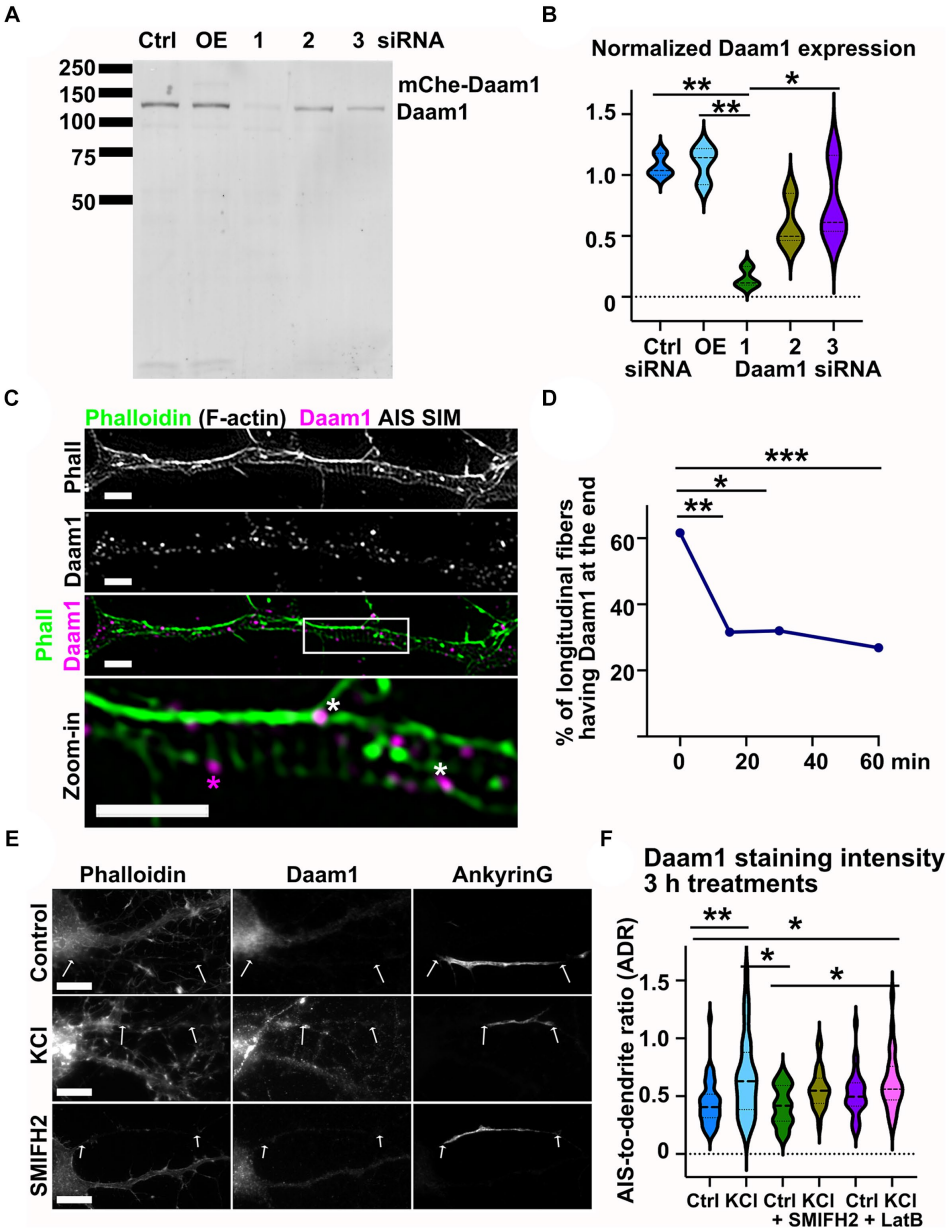
Daam1 localizes to the ends of longitudinal actin fibers in the AIS. **(A)** Anti-Daam1 antibody western blot of B16F1 cell lysates shows that the antibody can distinguish Daam1 from other proteins. From left to right, control cells treated with control siRNA, cells over-expressing mCherry-Daam1, next 3 bands: cells treated with 3 different siRNAs against Daam1. **(B)** Daam1 band intensity was compared to total protein. Data presents 3 separate experiments. The first Daam1-targeted siRNA (middle lane) effectively reduces Daam1 expression. ^*^p < 0.05, ^**^p < 0.01 according to a one-way ANOVA and Tukey’s multiple comparison test **(C)** DIV14 rat hippocampal neurons were fixed and stained with phalloidin (F-actin), Daam1 antibody, and ankyrinG antibody. The AIS was identified by ankyrinG staining and all stainings were imaged with SIM. Daam1 puncta were observed at the ends of longitudinal actin fibers (white stars) and occasionally co-localized with actin rings (magenta star). Scale bar: 1 μm. **(D)** Longitudinal actin fibers were visually tracked and the presence of Daam1 at the ends was analyzed. In control conditions, 61.6% of fibers had Daam1 at one or both ends. SMIFH2 treatment (10 μM) significantly reduced this portion to 31.6%, 32.0, and 26.9 at the 15-, 30-and 60-min timepoints, respectively. Data was pooled from three experiments and the number of analyzed fibers was N_ctrl_ = 73, N_SMIFH2_15min_ = 38, N_SMIFH2_30min_ = 25, N_SMIFH2_60min_ = 67. ^*^p < 0.05, ^**^p < 0.01, ^***^p < 0.001 according to a Chi-squared test of equal or given proportions. **(E)** DIV14 rat hippocampal neurons were treated with either 15 mM KCl to induce chronic depolarization of neurons or with 15 mM NaCl as a control in the presence or absence of 10 μM SMIFH2. After 3 h, cells were fixed and stained with phalloidin to visualize F-actin (left column in panel), with Daam1 antibody to visualize Daam1 (middle column) and with ankyrinG antibody to visualize the AIS by ankyrinG enrichment (right column). AIS areas based on ankyrinG enrichment are marked with two arrows in each image. Scale bar: 10 μm. **(F)** The relative intensity of Daam1 was analyzed from the AIS and dendrites. In control neurons, Daam1 staining was less bright in the AIS than in dendrites resulting in AIS-to-dendrite ratio of 0.44 ± 0.03 (equal distribution to AIS and dendrites would result in a value of 1). KCl treatment increased the portion of Daam1 in the AIS and AIS-to-dendrite ratio increased to 0.66 ± 0.06. SMIFH2 and latrunculin B treatments inhibited the KCl-induced increase on Daam1 AIS-to-dendrite ratio. N_ctrl_ = 43, N_KCl_ = 37, N_SMIFH2_ = 29, N_SMIFH2 + KCl_ = 24, N_LatB_ = 30, N_LatB + KCl_ = 29. ^*^p < 0.05, ^**^p < 0.01 as determined by a Kruskal-Wallis test. Violin plots present the median and interquartile range (25th and 75th percentile).

The fact that formin inhibitor SMIFH2 reduced the number of longitudinal actin fibers suggests that one or more formin family proteins are responsible for polymerizing longitudinal actin fibers. Thus, we wanted to find a formin family protein which co-localizes with longitudinal actin fibers. In 2020, Hamdan et al. identified proteins present in the AIS using the Bio-ID technique (Hamdan et al., 2020). This study identified Daam1 from the formin family proteins. While Bio-ID screen results do not exclude the possibility that other formin family proteins play roles in the AIS, these results prompted us to start with Daam1. We tested three Daam1 antibodies and an antibody purchased from Novus Biologicals showed the most specific Daam1 detection in Western blotting. We used mouse B16F1 melanocytes for testing because in these cells we have higher transfection efficiency, allowing us to over-express proteins in detectable amounts (OE, Figure 8A) or effectively reduce Daam1 expression by Daam1-targeted siRNA (1, 2, 3 siRNA, Figure 8A). Thus, by using cell lysates from Daam1 knock-down cells as controls, we show which antibody detects specifically Daam1 in Western blot as band intensities reduce upon Daam1 knockdown (Figure 8A). As determined by western blotting, the best Daam1 siRNA reduced the expression of Daam1 to 15% of its endogenous expression (Figure 8B). We then stained DIV14 hippocampal neurons with phalloidin to visualize actin filaments, anti-Daam1 antibodies to visualize endogenous Daam1, and anti-ankyrinG antibodies to visualize the AIS. These stainings revealed that Daam1 often localized to the ends of longitudinal actin fibers (Figure 8C). We further tested whether the formin inhibitor SMIFH2 affects Daam1 localization. A short 15-min treatment already reduced the localization of Daam1 to the ends of longitudinal fibers (Figure 8D).

As Daam1 localized to the ends of longitudinal actin fibers, we tested whether its general localization changes in neurons upon KCl treatment (Figure 8E). Analysis showed that Daam1 localization in the AIS area was increased upon KCl addition (Figure 8F). Changes in axon width could possibly affect the intensity of Daam1 in axons, but we confirmed already in earlier experiments that KCl treatment did not affect axon width (Figure 7E).

Taken together, Daam1 localization suggests that it could be a polymerizing factor for longitudinal actin fibers.

## Discussion

Alterations in AIS structure contribute to the pathophysiology of various psychiatric, neurodevelopmental, neurodegenerative, and autoimmune diseases, as well as brain trauma or injury (Garrido, 2023). Since the discovery of AIS plasticity, a number of diseases have been linked to changes in AIS length or position. Tau dysfunction and amyloid-β plaques both disrupt the AIS and alter its length and position (Marin et al., 2016; Hatch et al., 2017; Antón-Fernández et al., 2022), and the AIS cytoskeleton is destabilized in Alzheimer’s disease patients (Sohn et al., 2016). Further, the absence of AIS structural plasticity was demonstrated in a frontotemporal dementia mouse model (Sohn et al., 2019). Thus, elucidating the basic processes underlying these modifications in AIS position could improve our understanding of disease pathologies.

**Figure 9:**
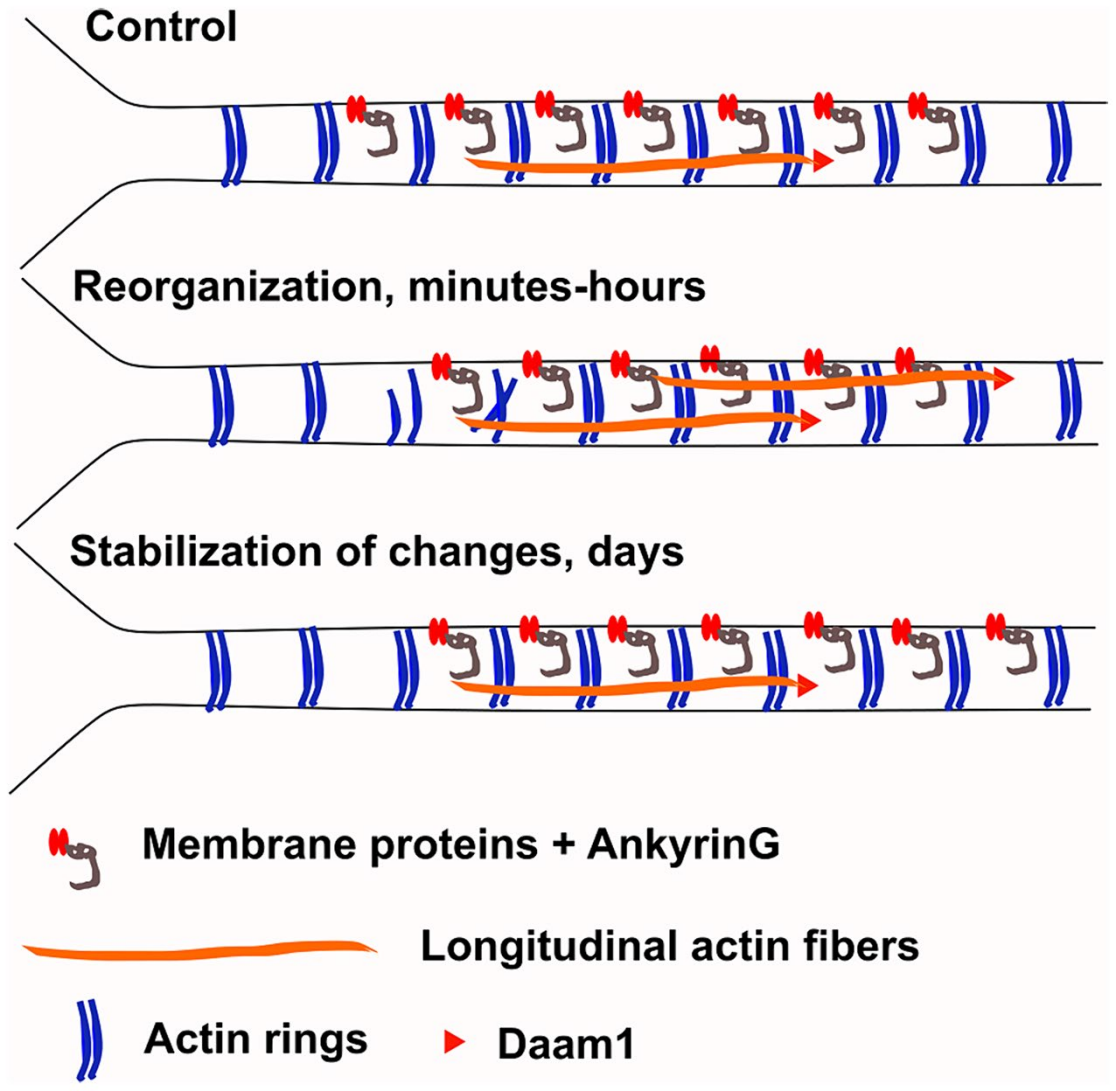
Working model. We hypothesize that changes in AIS plasticity can be broken up to 3 steps: 1) destabilization of actin rings, allowing higher mobility of AIS proteins, 2) translocation of transmembrane and other AIS proteins and 3) then re-stabilization of the structure. Our results support the hypothesis that actin rings are disrupted at proximal end of the AIS allowing release of membrane bound proteins. This result could explain why ankyrinG was released from the proximal site of AIS after three-hour KCl-treatment. The number of longitudinal actin fibers increase throughout the AIS and formin family protein Daam1 localized to the ends of longitudinal actin fibers suggesting that it could serve as a polymerizing factor for these structures. However, the role of longitudinal actin fibers in AIS plasticity is unclear. They could play a role in protein transport or they could facilitate endocytosis of transmembrane proteins. The AIS actin cytoskeleton re-organizes in minutes and hours (Re-organization, middle). After 2 days of chronic depolarization, the AIS actin cytoskeleton was indistinguishable from control conditions and ankyrinG had moved distally (Stabilization, below).

While AIS plasticity is important for proper functionality of AIS-containing neurons, the cellular and molecular mechanisms underlying AIS plasticity are poorly understood. Here, we analyzed changes in the AIS actin cytoskeleton during AIS structural plasticity. We showed that the number of longitudinal actin fibers increased upon plasticity induction and reverted back to control levels when plastic changes were complete (Figure 1). We further showed that actin polymerization, in particular formin-mediated actin polymerization, is required for AIS plasticity (Figures 4, 6). From the formin family of proteins, Daam1 localized to the ends of longitudinal actin fibers (Figure 8). These results indicate that active polymerization of actin filaments is required for proper AIS plasticity. We also observed modest changes in actin ring periodicity upon KCl-induced depolarization (Figures 5, 7). The fact that these changes occurred in the presence of latrunculin B or SMIFH2 suggest that these changes did not require actin polymerization. Since both latrunculin B and SMIFH2 also blocked shortening of the AIS at 3 h, changes in periodicity are not sufficient for AIS plasticity, but it may be a necessary step. This suggests that changes associated with AIS plasticity can be broken up into 3 steps: destabilization of rings allowing higher mobility of AIS proteins, translocation of transmembrane and other AIS proteins, and then re-stabilization of the structure (Figure 9). Thus, latrunculin B or SMIFH2 treatment would allow destabilization of rings but further steps would be halted because of blocked actin polymerization. Possible mechanisms to affect ring periodicity are actin depolymerization and degradation of rings or altered interactions with proteins which regulate the distance of ring periodicity, such as spectrin or myosin II.

Our working hypothesis is that changes in the actin cytoskeleton during AIS plasticity would have a similar role to synaptic plasticity-induced changes in dendrites or dendritic spines. Upon high frequency stimulation, the actin cytoskeleton in dendritic spines is first dissolved and then quickly re-polymerized and stabilized (Hlushchenko et al., 2016). These changes occur in minutes. The purpose of re-organization of the actin cytoskeleton is that it allows addition or replacement of excitatory synapse receptors. In dendrites, synaptic activation led to a change from actin rings to longitudinal actin fibers in 10 min (Lavoie-Cardinal et al., 2020). While the exact purpose of this change is unclear, we speculate that this increase in dynamic activity in the actin cytoskeleton facilitates or allows other plastic changes to occur. Axonal actin rings are considered to be very stable structures and have previously been shown to not change in the same way as dendritic rings (Lavoie-Cardinal et al., 2020).

We hypothesized that the AIS actin cytoskeleton dynamically re-organizes upon neuronal activation, similar to dendritic spines and dendrites, it just occurs slower. Based on earlier results (Lavoie-Cardinal et al., 2020), we expected that chronic depolarization would increase the number of longitudinal actin fibers and disrupt actin rings (Figure 9). Actin rings can trap ion channels and other membrane proteins between them (Albrecht et al., 2016; Rentsch et al., 2024) so dissolving actin rings from one area may release ion channels and allow them to move to another area. Thus, changes in actin rings could allow changes in membrane protein location. Since 3-h KCl-treatment shortens the AIS from the proximal end, we hypothesized that changes in the actin cytoskeleton take place primarily in the proximal end of AIS. We measured actin ring periodicity in KCl-treated neurons and observed a small change in periodicity (Figures 5B, 7D). Latrunculin B and SMIFH2 treatments improved the clarity of periodicity and with these treatments the effect with KCl was clear. In latrunculin B treated cells, we could observe a significant reduction in periodicity and autocorrelation amplitude especially in the proximal end of AIS after 3-h KCl-treatment, supporting our original hypothesis that disruption of actin rings at the proximal end would release membrane bound proteins (Figures 5, 9).

It is important to note that although changes in periodicity were relatively modest, even small changes can affect the functionality of the AIS. In our earlier study, inhibition of tropomyosin resulted in visible changes in 6 h, but we detected significant attenuation of firing frequency already after 15 min (Abouelezz et al., 2020). Thus, small, “invisible” changes can be functionally significant.

The main visible change in the actin cytoskeleton at the 3 h-time point was an increase in the number of longitudinal actin fibers. Longitudinal actin fibers are relatively novel structures and their function in neurons is unclear. We showed that formin-dependent polymerization is necessary for AIS plasticity, but it is not clear whether the appearance of longitudinal actin fibers is necessary. To test the hypothesis that changes would be biggest in the proximal end of the AIS, we analyzed the number of longitudinal actin fibers at different locations along the AIS. However, increase in the number of longitudinal actin fibers was relatively equally distributed along the AIS. These results suggest that while disruption of actin ring periodicity is occurring locally at proximal end of the AIS, the increase in longitudinal actin fibers does not show any specific site of origin in the AIS. This is possibly because their dynamic nature results in them rapidly spreading through the AIS before our measurement timepoint.

From the formin family of proteins, Daam1 localized to the ends of longitudinal actin fibers. Daam1 is a formin family protein which polymerizes actin filaments using a processive capping technique similar to other formins (Jaiswal et al., 2013). In addition, Daam1 bundles actin filaments (Jaiswal et al., 2013). Daam1 has a punctate localization in various cells. Puncta may be on actin filaments (bundling) (Jaiswal et al., 2013), on the plasma membrane (Luo et al., 2016) or on endocytic vesicles (Kida et al., 2007). Similarly to many other formins, Daam1 is folded in closed, inactive conformation and this fold can be opened by binding to other proteins, such as Dishevelled (Liu et al., 2008). We observed Daam1 primarily at the end of longitudinal fibers, therefore it appears to be involved in polymerization, not bundling. Further, formin inhibition reduced the number of longitudinal actin fibers, not the thickness of fibers. While we found Daam1 localizing to the end of fibers, there are likely also other formins which take part in polymerizing longitudinal actin fibers. One of them could be mDia1, which was shown to be necessary for AIS maintenance (Zhang et al., 2021). The localization of other formin family proteins should be analyzed in up-coming studies.

We wanted to test whether Daam1 is necessary for the longitudinal actin fibers using Daam1-siRNAs. However, this was technically challenging as SIM imaging of such thin actin fibers requires low density neuronal cultures, where axons can be separated and identified clearly from other neurites. In contrast, siRNA transfection requires high-density neuronal cultures to maintain neurons´ good health during the transfection. Therefore, we could not get conclusive results from these experiments.

In addition to localizing to the ends of longitudinal actin fibers, Daam1 sometimes co-localized with actin rings (Figure 8C). This indicates that Daam1 could play a role also in actin ring assembly. However, formin activity was not necessary for actin ring periodicity (Figure 7D) suggesting that formins are not necessary for the maintenance of actin rings.

Actin polymerization in AIS plasticity could have also other functions beyond just changing actin structures that maintain the AIS structure. Recent studies have shown the involvement of endocytosis in the assembly, maintenance, and plasticity of the AIS (Torii et al., 2020; Eichel et al., 2022; Fréal et al., 2023). The common message from these studies is that 1) reducing endocytosis during AIS assembly stabilizes AIS structure, 2) some level of endocytosis is, however, required to maintain AIS structure and 3) short-term AIS plasticity (30 min) induction by NMDA application increases endocytosis and shortening of the AIS. The activity of actin regulating proteins myosin II and calcineurin is required for AIS plasticity (Evans et al., 2013, 2017). Myosin II contractility and calcineurin activity are involved in many cellular processes, including endocytosis. Similarly, proper actin dynamics are required for proper endocytosis, with known roles for Daam1 in endocytosis (Kida et al., 2007). While these earlier results suggest that endocytosis could also underly KCl-induced AIS plasticity, blocking endocytosis did not block KCl-induced AIS plasticity at three hours (Evans et al., 2017). Inhibiting endocytosis with the dynein inhibitor Dynasore shortened the AIS length but it did not affect KCl-induced shortening of the AIS (Evans et al., 2017). These results oppose the NMDA-study, in which Dynasore inhibited shortening of the AIS in 30 min (Fréal et al., 2023). The most plausible explanation is that different AIS plasticity induction methods utilize different cellular processes.

Although we cannot provide the final mechanistic explanation for the role of the actin cytoskeleton in AIS plasticity, this study brings many new pieces to the puzzle and highlights the importance of actin dynamics in the process. Hopefully these pieces help us to understand the molecular mechanisms of AIS plasticity in the near future.

## Materials and methods

### Neuronal cultures and transfections

Neuronal cultures were prepared as in Hotulainen et al. (2009) and Abouelezz et al. (2020). Dissection and dissociation of neurons was done by the Neuroscience Center (HiLIFE, University of Helsinki). Hippocampi were dissected from embryonic day 16–17 Wistar rat fetuses. Neurons were dissociated in 0.05% papain and triturated in Ca^2+^- and Mg^2+^-free HBBS medium with 1 mM sodium pyruvate and 10 mM HEPES (pH 7.2). The hippocampus has many different cell types and when preparing hippocampal primary neuron cultures, we plate them all. In our analyses we focus on excitatory neurons, which we select based on their morphology. We have tested with antibody stainings that cultures have, in addition to excitatory neurons, inhibitory neurons (Gad65/67-staining) and astrocytes (GFAP-staining). In experiments, these cell types are relatively easy to distinguish from excitatory neurons based on their morphology.

Cells were plated on 13 mm glass coverslips (VWR) or 13 mm high-precision glass coverslips for SIM (Marienfeld) in 24-well plates (Corning) coated with 0.01 mg/mL poly-L-lysine (Sigma-Aldrich) in Neurobasal medium (Invitrogen) with B-27 (Invitrogen), L-Glutamine (Invitrogen), and primocin (Invivogen). Cells were plated at a density of 50,000 cells/cm^2^ for all experiments except those using SIM microscopy. For SIM experiments, cells were plated at 10,000/cm^2^. Cells were cultured in a humidified incubator at 37°C with 5% CO_2_, with media refreshed twice/week at regular intervals. Transfections were performed on DIV10-12 neurons using Lipofectamine 2000 (Invitrogen) as in Hotulainen et al. (2009) using 0.7 μg plasmid/well in 24-well plates. For siRNA transfections, RNAiMAX (Invitrogen) was used with 30 nM siRNA construct. For high-KCl conditions, KCl was added directly to the cell culture media to a final concentration of 15 mM with the same concentration of NaCl used as a control. The formin inhibitor SMIFH2 (Tocris Bioscience; 4,401) and actin monomer sequestering drug Latrunculin B (SigmaAldrich; L5288) were used concentrations of 10 μM and 5 μM, respectively, with DMSO used as a control.

### Immunostaining

Cells were fixed in 4% paraformaldehyde in PBS for 12 min at room temperature and permeabilized in 0.05% Triton-X in PBS for 10 min. Blocking and washing between steps were done with 0.2% bovine serum albumin in PBS (BSA-PBS). Primary antibodies were incubated at room temperature in BSA-PBS for 90 min, except for Daam1 which was incubated overnight at 4°C. All secondary antibodies were incubated at room temperature in BSA-PBS for 60 min. Phalloidin was incubated at room temperature for 2 h. Coverslips were mounted on glass slides using ProLong Gold (ThermoFisher). The complete protocol for staining for SIM experiments can also be found in Micinski et al. (2022).

Mouse monoclonal anti-Ankyrin G antibody (1:500; clone 106/36) was purchased from NeuroMab. Rabbit polyclonal anti-Daam1 antibody (1:200) was purchased from Novus Biologicals (NBP1-81492). Alexa488-conjugated phalloidin (1:50 for SIM imaging, 1:400 for all other experiments) was purchased from ThermoFisher (A12379). Alexa-conjugated secondary antibodies Alexa-647 (1:500) and Alexa-568 (1:500) were purchased from ThermoFisher (Alexa647: A-21236; Alexa568: A-11036) while Alexa-555 (1:500) was purchased from Abcam (ab150078). siRNA against rat Daam1 was purchased from GenePharma (NM_001108030, sequence #1 5’-GCUGUAUAAAGGCACUAAUtt-3′, sequence #2 5’-GCUGCACUAUCAGAAGUAUtt-3′, sequence #3 5’-CAGCCUGUUAUAGAUAAAUtt-3′). mCherry-Daam1 was a kind gift from Maria Vartiainen (University of Helsinki, Finland).

### Western blotting

Three different Daam1-siRNAs were tested in B16F1 cells cultured on 6-well plates. Following transfection, cells were lysed with radio immunoprecipitation assay (RIPA) buffer containing 50 mM Tris–HCl pH 7.4, 150 mM NaCl, 1 mM EDTA, 0.25% sodium deoxycholate and 1% NP-40, 1% SDS, and a phosphatase/protease inhibitor cocktail (Roche). A BCA protein assay (ThermoFisher) was used to get protein concentrations and 20 μg of protein/sample was run through a 10% SDS-PAGE gel. Protein was transferred to a polyvinylidene difluoride (PVDF) membrane and blocked using EveryBlot Blocking buffer (Bio-Rad #12010020). The membrane was incubated overnight at 4°C with anti-Daam1 primary antibody (mouse, 1:500, Novus Biologicals), washed 3 × 10 min in TBS-0.1% Tween 20 (TBS-T), then incubated in StarBright Blue 520 (1:2500, Bio-Rad, #12005866) for 1 h at room temperature. Primary and secondary antibodies were both diluted in EveryBlot Blocking buffer. After washing 3 × 10 min in TBS-T again, the membrane was imaged on a ChemiDoc MP imaging system (Bio-Rad). Daam1 protein levels were quantified against the total protein/lane using Image Lab software (Bio-Rad). The protein ladder used was the Precision Plus Protein Unstained Protein Standard (Bio-Rad, #161–0363).

### Imaging

For SIM, we used a DeltaVision OMX SR system (GE Healthcare Life Sciences) with a 60× 1.42 NA PlanApo N oil-immersion objective. We used AcquireSR software for acquisition and SoftWoRx for image reconstruction and alignment for both fixed and live-cell SIM. For diffraction-limited epifluorescence imaging we used a Zeiss AxioImager Z2 (Zeiss) with a 63× 1.4 NA oil-immersion objective and a Hamamatsu Orca Flash 4.0 LT camera (Hamamatsu) and the Zeiss Zen software for acquisition.

### Image analysis

We used Fiji software for all image analysis (Schindelin et al., 2012). All analyses were done blinded to the experimental condition.

To analyze actin ring periodicity, we plotted four line profiles of approximately 2 μm each along the AIS in every image, avoiding patches and fasciculations. We used MATLAB (MathWorks Inc) to locally normalize the fluorescence intensity of each line profile with “movmean,” “autocorr” to calculate the autocorrelation function for each profile, and “findpeaks” to detect individual peaks for each profile to calculate the inter-peak distances. This procedure is described previously in Abouelezz et al. (2020). The amplitude of the first peak around 200 nm was calculated by taking the highest measured point of the peak and first two lowest values presenting the first two valleys around the first peak. The average of the two lowest values was subtracted from highest value. A few functions which did not show a peak around 200 nm were omitted from analysis.

To quantify the number or length of longitudinal actin fibers, SIM images were taken of the AIS, prioritizing neurons with few/no other neurites in close proximity to the AIS. Max projections were made of background-subtracted z-stacks. Line profiles were drawn over all visually identified filaments within the images, and these profiles were checked against the original z-stacks to ensure identified filaments were intracellular and not overlapping neurites/filopodia. Only longitudinal actin fibers which were clear in SIM images with original brightness were included to analysis. If it was unclear whether there were two fibers after each other, it was estimated that they are one continuous fiber. All counted or measured fibers were at least 1 μm in length. Angular variation from the AIS shaft was a maximum of 45°, usually far less. Greater than 45° usually indicated that the fiber was extracellular, and merely overlapping the AIS in the image.

To quantify the AIS-to-dendrite ratio (ADR) of Daam1, the same line profiles drawn for the analysis of AIS position were used to define the AIS. New line profiles of at least 50 μm starting from the soma were drawn for dendrites. ADR was calculated as the mean fluorescence intensity of Daam1 within the AIS divided by the mean fluorescence intensity of Daam1 in the first 50 μm of all dendrites of the same neuron.

### Analysis of AIS position

Line profiles were drawn using the Fiji plugin SNT^2^ starting from the soma and extending beyond the Ankyrin G staining in the axon. Analysis was performed as in Grubb and Burrone (2010) and Van Beuningen et al. (2015) using the same MATLAB scripts referenced above (Van Beuningen et al., 2015) (see Footnote 1). Line profiles were smoothed using a 3 μm rolling average around the pixel of interest, then normalized using the minimum and maximum smoothed fluorescence to values between 0 (minimum) and 1 (maximum). The AIS start and end positions were determined to be where these normalized values first fell to 0.33 on either side of the maximum, regardless of whether they increased above 0.33 farther down the line profile.

To quantify the mean axon width, line profiles were drawn perpendicularly across AISes near the center of the ankyrinG staining. The same MATLAB script from above (Van Beuningen et al., 2015) was used, without smoothing, to distinguish the axon from the background. The cutoff was set to be when ankyrinG fluorescence intensity values first fell to 33% of the maximum fluorescence intensity in the line profile.

### Statistical analyses

All tests were performed using either MATLAB (MathWorks Inc) or R (R Foundation for Statistical Computing) with RStudio Desktop (Posit PBC). Graphs were created in MATLAB and GraphPad Prism (GraphPad Software Inc). All data were checked for normality with the Shapiro–Wilk test and for homogeneity of variance using Levene’s test. For all parametric data we used either the two-sample *t*-test or one-way ANOVA. For non-parametric data, we used the Kruskal-Wallis test or the Mann–Whitney U test. The Tukey–Kramer test was used for multiple comparisons. For comparing the distributions of inter-peak distances under different experimental conditions, the Kolmogorov–Smirnov test was used. All relevant groups were statistically compared to each other but only significant changes are marked to plots.

## Data availability statement

The original contributions presented in the study are included in the article/supplementary material, further inquiries can be directed to the corresponding author.

## Ethics statement

Ethical approval was not required for the study involving animals in accordance with the local legislation and institutional requirements because we dissociate neurons from rat embryos but rats are euthanized before any procedures. According to Finnish law we can do this without ethical approval.

## Author contributions

DM: Conceptualization, Data curation, Formal analysis, Investigation, Methodology, Software, Validation, Visualization, Writing – original draft, Writing – review & editing. PH: Conceptualization, Funding acquisition, Project administration, Resources, Supervision, Validation, Visualization, Writing – original draft, Writing – review & editing.

## Footnotes

^1^https://github.com/ekatrukha/AIS

^2^https://imagej.net/plugins/snt/
